# The effects of submaximal exercise on acid–base balance in people with long COVID

**DOI:** 10.1113/EP093996

**Published:** 2026-11-14

**Authors:** Mark A Faghy, Callum Thomas, Rebecca Owen, Ruth EM Ashton, Szabolcs Pallagi, Marie J Grigg, Thomas Bewick, David Broom, Darren Richardson, Lewis A. Gough

**Affiliations:** ^1^ School of Sport, Exercise and Health Sciences Loughborough University Loughborough UK; ^2^ School of Health, Sport and Rehabilitation University of Derby Derby UK; ^3^ Research Centre for Healthcare and Community Transformation Coventry University Coventry UK; ^4^ University Hospitals of Derby and Burton NHS Foundation Trust Derby UK; ^5^ Institute for Health and Care Improvement York St John University York UK; ^6^ Research for Human Performance and Health Laboratory Birmingham City University Birmingham UK

**Keywords:** acid–base disturbance, diagnostic mechanisms, long COVID, pathophysiology

## Abstract

Better pathophysiological understanding of long COVID (LC) is required, and disruption of acid–base regulation has been proposed as a potential mechanism underlying exercise intolerance observed in LC; however, empirical evidence remains limited. Accordingly, this study aims to systematically characterise day‐to‐day variation in acid–base status in individuals living with LC. Thirty people (19 female) with a confirmed diagnosis of LC or 15 healthy controls (CON; 8 female) completed three study visits. Participants completed two submaximal cardiopulmonary exercise tests (CPETs) separated by 24 h. Pre‐ and post‐exercise fingertip capillary blood samples were collected on both CPET days for acid–base balance (pH, HCO_3_^−^, anion gap, K^+^, Na^+^, Cl^−^ and ionized calcium (iCa^2+^)) and lactate. Subgroup analysis was conducted for those presenting with metabolic acidosis between LC and CON. Whole group analysis revealed no differences between LC and CON for acid–base balance (*P *> 0.05) or CPET variables (*P *> 0.05). However, a subgroup analysis determined that following CPET1, 17% (*n* = 5/30), and following CPET2, 33% (*n* = 10/30) of those with LC demonstrated metabolic acidosis, with small to large effect sizes (0.20 → 0.80) between LC and CON for pH, HCO_3_^−^, anion gap, K^+^, Na^+^, Cl^−^, iCa^2+^ and lactate. For CON, metabolic acidosis was identified for 6% (*n* = 1/18) on CPET day 1 and 11% (*n* = 2/18) on CPET day 2. Our findings suggest that some people with LC may experience acid–base balance disturbances following repeated submaximal exercise. Accordingly, a pathological subgroup is hypothesised, and these findings should provoke independent validation.

## Abstract (highlights)

- **What is the central question of this study?**
  Does acid–base balance disturbance occur in people living with long COVID and how does this compare to a control group?
- **What is the main finding and its importance?**
  Submaximal two‐day CPET observed no whole group disturbances in acid–base balance between those with long COVID and controls. However, an exploratory, hypothesis generating analysis identified that 17% and 33% of the long COVID cohort demonstrated disturbed acid–base balance compared to the control group post‐CPET day 1 and day 2. Future research should independently verify these observations.

## 1 INTRODUCTION

Long COVID is a post‐viral condition that represents a collection of post‐acute and long‐term health impacts that can be caused following a single infection with SARS‐CoV‐2. Long COVID is complex with multiple underlying pathologies that present as a broad and potentially disabling symptom profile consisting of over 200 symptoms that fluctuate in frequency and intensity. It is usually diagnosed following 12 weeks (or longer) of symptoms from the initial SARS‐CoV‐2 infection (Owen et al., 2025). Long COVID affects more than two million people in the United Kingdom, and it is estimated that more than 400 million individuals are impacted by long COVID globally (Al‐Aly et al., 2024; Greenhalgh et al., 2024). Currently, there are no confirmed treatments for long COVID, and whilst potential options are being tested to determine their efficacy, there is a need to inform the development of efficacious management strategies to improve the quality of life (QoL) and functional status of people living with long COVID.

Increasing the mechanistic and pathophysiological understanding of long COVID and other post‐viral illnesses is imperative to developing effective treatment and management strategies. However, a key challenge is the prevalence of post‐exertional malaise (PEM) (also often called post‐exertional symptom exacerbation (PESE)), a highly prevalent response to exertion that affects almost half of long COVID patients (Thaweethai et al., 2023). PEM is characterised by the worsening of existing symptoms or the emergence of new symptoms following physical, mental or cognitive exertion. Symptoms typically begin within 48 h of exceeding an individual's PEM threshold and may persist for days or even months (Thaweethai et al., 2023). Emerging hypotheses suggest that PEM may be underpinned by disordered metabolic responses to exertion, including alterations in acid–base balance that impair recovery and exacerbate symptom burden (van de Togt and Rossman, 2023). Therefore, the use of novel approaches to assessing physical function is needed to ensure that the diagnostic and mechanistic evidence base can be determined. Cardiopulmonary exercise tests (CPET) are commonplace in health research to detect abnormal responses to physical stimuli and can offer an objective assessment of functional capacity (Faghy et al., 2020, 2024). However, the use of maximal protocols has the potential to exacerbate symptom profiles and carry an increased level of risk of harm and worsening symptoms. The safety and utility of using submaximal protocols that are prescribed relative to a patient's current functional status can determine abnormal physiologic responses, as documented elsewhere (Faghy et al., 2024; Thomas et al., 2026). This is important as alterations in acid–base balance have been proposed as a potential mechanism underlying reduced exercise performance in long COVID (van de Togt and Rossman, 2023).

Whilst disruption of acid–base balance has been reported in patients with acute COVID infection (Alfano et al., 2022; Sjöström et al., 2021; Chiumello et al., 2022), there remains a limited understanding of how these regulatory mechanisms operate in people living with long COVID, both at rest and during physical activity. In healthy individuals, acid–base homeostasis is closely integrated with exercise physiology, with increasing exercise intensity leading to greater glycolytic flux, lactate and hydrogen ion production, and a consequent reliance on buffering and ventilatory responses to maintain pH and sustain muscle function. In contrast, emerging evidence suggests that individuals with long COVID may exhibit altered metabolic responses to exercise. To date, few studies have examined post‐exercise lactate responses in this population, which can be an appropriate proxy marker for acid–base balance; however, those available report elevated lactate concentrations at both maximal (de Boer et al., 2022) and submaximal intensities (Thomas et al., 2026). This pattern indicates an earlier reliance on anaerobic metabolism that might be due to reduced mitochondrial oxidative capacity and an increase in overall cardiovascular strain, and thus, a higher perception of effort for the same given workload. Evidence to date would support this notion via the well‐documented impairments in aerobic capacity observed in long COVID (Lien et al., 2019). Such metabolic alterations are likely to have important consequences for acid–base regulation during exertion, potentially contributing to exercise intolerance and symptom exacerbation. However, Lien et al. (2019) did not assess lactate‐associated metabolic or acid–base markers, limiting insight into the mechanistic links between disordered energy metabolism and physiological stress responses in long COVID.

Based on this evidence, it is conceivable that acid–base balance disturbances may occur during exercise in people with long COVID; however, this has not yet been assessed empirically. Evidence of acid–base balance disturbances following exercise in individuals with long COVID may inform targeted management strategies to mitigate these complications (e.g., alkalotic buffers), thereby improving symptom burden, functional status and QoL. Accordingly, we aimed to profile changes in acid–base balance in people living with long COVID using submaximal CPET protocols to advance pathological understanding, characterise symptom responses, and inform the development of innovative management strategies that could improve patient outcomes.

## 2 METHODS

### 2.1 Participants

Following ethics approval from the host institution (Derby University, UK; ETH2324‐5420) and volunteers providing written informed consent, 30 individuals with a confirmed long COVID diagnosis were recruited (*n* = 19 females, age 48 ± 12 years, height 169 ± 8 cm and body mass 84 ± 15 kg; Table 1) via convenience sampling. The study conformed to the standard set by the *Declaration of Helsinki*, except for registration in a database. Patients were recruited through participation in previous long COVID research, word of mouth and social media. Fifteen people (*n* = 8 female, age 41 ± 11 years, height 169 ± 9 cm and body mass 79 ± 11 kg) were recruited to comprise a control group (CON). Patients were screened for PEM using the DePaul symptom questionnaire (DSQ; Cotler et al., 2018) before attending three study visits. Fifteen age‐ and sex‐matched controls without long COVID were also recruited (Table 1).

**TABLE 1:** Demographic data of long COVID and control groups.

Variable	Long COVID (*n = 30*)	Control (*n = 15*)
Sex		
Male	11	7
Female	19	8
Age (years)	49 ± 11	41 ± 11
Ethnicity		
White British	29	
Asian British	1	
Weight (kg)	84 ± 15	79 ± 11
Height (cm)	169 ± 8	169 ± 9
Pre C‐19 Health Rating	1 ± 1	2 ± 1
Current Health Rating	4 ± 1	2 ± 1
Number of confirmed C‐19 infections	2 ± 1	2 ± 1
Number of Suspected C‐19 Infections	3 ± 1	3 ± 1

Data presented as means ± standard deviation.

Abbreviation: C‐19, Covid‐19.

### 2.2 Screening procedures

Long COVID participants were screened via a two‐stage process to confirm eligibility. Initial eligibility was confirmed via telephone consultation, which required evidence of a previous COVID‐19 infection and a clinical long COVID diagnosis. Individuals were also required to have sufficient English‐language comprehension and cognitive ability to understand the study protocol, provide informed consent and follow instructions. Exclusion criteria included <18 years of age, having been admitted to or received treatment from intensive care units, having an unconfirmed COVID‐19 test or no retrospective clinician diagnosis, or having no confirmed long COVID diagnosis from a healthcare professional. Individuals were also excluded at this stage of recruitment if they were at high risk of moderate‐to‐severe PEM following administration of the DSQ (Cotler et al., 2018).

Eligibility was confirmed in 30 of 38 (79%) people with long COVID, and these individuals were invited to a detailed screening visit. Reasons for exclusion at the point of eligibility were due to high risk of PEM, issues with mobility preventing completion of exercise tests and recent (<3 months) infection with SARS‐CoV‐2. Detailed screening included full demographic profile (age, sex, ethnicity, smoking history and past medical history), detailed acute COVID‐19 history (number of confirmed infections, vaccinations, hospital admissions, acute symptoms, retrospective assessment of performance status, pre‐infection exercise tolerance), long COVID (diagnosis, symptoms, current access to treatments/services) and confirmation of PEM risk via re‐administration of the DSQ. Following confirmation of eligibility, participants completed three experimental sessions, including a baseline assessment (day 1) and two submaximal CPET visits separated by 24 h (days 7 and 8). Control participants were screened for COVID‐19 infections, vaccinations and current health status only, and data are summarised in Table 1.

### 2.3 Baseline assessment

Baseline data collection included physiological observations, including heart rate (HR), blood pressure (BP), peripheral oxygen saturation ($S_{pO_{2}}$), respiratory rate, and temperature. Patient‐reported outcomes (PROMS) were administered digitally via RedCap (Vanderbilt University, Nashville, TN, USA). Where requested, PROMS were also available in paper form and manually entered into RedCap by the research team. All PROMS were checked for completeness at the start of subsequent visits, and participants were allowed to seek clarification, ask questions and complete any outstanding questions. The DSQ was administered at all sessions as described previously. QoL was assessed using the EuroQol 5‐Dimension 5‐Level (EQ‐5D‐5L), which is routinely used in the assessment of QoL in respiratory research and includes a visual analogue score (VAS) of 0–100, 0 being the worst health you can imagine and 100 being the best health you can imagine (EQ‐5D‐5L; Herdman et al., 2011). Fatigue was assessed via two separate measures of fatigue: (1) the Fatigue Assessment Scale (FAS), a self‐reported questionnaire validated to assess the presence and severity of fatigue (De Vries et al., 2004); and (2) the modified fatigue impact scale (MFIS), a 21‐item self‐reported questionnaire assessing fatigue across the physical, cognitive and psychosocial domains (Larson, 2013). Functional status was assessed via the 6‐min walk test (6MWT) and conducted according to published guidelines (Agarwala and Salzman, 2020). Data from the 6WMT were used to determine the initial work rate (WR) for an adapted submaximal CPET protocol that is tolerated and safe for long COVID patients (Thomas et al., 2026). Starting WR was based on 6MWT distance; strata I: 6MWT distance <350 m (starting WR of 10 W); with subsequent increments of 5 W), strata II: 6MWT distance 350–400 m (starting WR of 20 W with subsequent increments of 5 W) and strata III: 6MWT distance >400 m (starting WR of 30 W with subsequent increments of 5 W).

### 2.4 CPET

Participants completed two submaximal CPETs (CPET1 and CPET2) on a friction‐loaded cycle ergometer (Lode Excalibur Sport, Groningen, The Netherlands) in a thermoneutral environment separated by 24 h. Full protocol details have been published elsewhere (Thomas et al., 2026). Briefly here, the protocol began with a 3‐min rest period, followed by a maximum of 12 min of exercise delivered via a stepwise incremental protocol. Pre‐ and post‐exercise capillary blood samples were collected from the distal phalanx of the participants' preferred hand and finger and analysed to determine acid–base status (Radiometer, ABL9, Crawley, UK) and blood lactate (Lactate Plus, Nova Biomedical, Runcorn, UK). Acid–base variables pH, bicarbonate (HCO_3_^−^), potassium (K^+^), sodium (Na^+^), ionized calcium (iCa^2+^), and chloride (Cl^−^), arterial partial pressure of oxygen ($P_{aO_{2}}$), and arterial partial pressure of carbon dioxide ($P_{\mathrm{aC}O_{2}}$) were measured, and the anion gap was calculated as: (Na^+^ + K^+^) − (Cl^−^ + HCO_3_^−^). Expired respiratory gases and ventilatory profile were assessed using online breath‐by‐breath analysis (Cortex Metalyser, 3B, Leipzig, Germany), and a 12‐lead electrocardiogram (ECG, Custo Cardio 300 BT, Ottobrunn, Germany) were analysed continuously. Rating of perceived exertion (RPE; 6 to 20 scale) was measured during the final 15‐s period of each minute. All data were exported for offline analysis using Microsoft Excel.

### 2.5 CPET data processing

Briefly here, raw gas analysis data were exported in 30‐s moving averages and middle 5‐of‐7 breath averages to reduce variability and identify data outliers or missing data. The middle 5‐of‐7 breaths data were used to determine the ventilatory threshold (i.e., VT1) and respiratory compensation point (i.e., VT2) using the V‐slope method (Pescatello, 2014). The 30‐s moving‐average data were used for baseline, peak and iso‐time peak calculations whereby iso‐time peak was defined as WRs that were matched within participants for total duration of cycle exercise (comparison of the shortest duration reached of the two datasets) (Curtis et al., 2015; Nicolò et al., 2021). Data are presented as means ± standard deviation.

### 2.6 Statistical analysis

Statistical analysis was conducted using a software package (JASP, Version 0.19, Amsterdam, The Netherlands). Distribution of data was assessed using the Shapiro–Wilk test and visual inspection of Q‐Q plots. In the whole group analysis, a 2 × 4 mixed‐design analysis of variance (ANOVA) was conducted on the acid–base balance variables to examine treatment groups (i.e., LC and CON) across measurement occasions. Treatment was the between‐participant factor and measurement occasion was the within‐participant factor. The measurement occasion (referred to as *time*) comprised day 1 pre‐exercise (rest), day 1 post‐exercise, day 2 pre‐exercise (rest) and day 2 post‐exercise. The repeated‐measures factor accounted for the dependence between observations obtained from the same participant across measurement occasions. Analysis was made for main effect for time or treatment, and the treatment × time interaction. Sphericity was assessed via the Epsilon value with the required adjustment made where necessary (i.e., Greenhouse–Geisser). Where main effects for time or treatment × time interactions were observed, the *P*‐value is reported along with the Partial eta squared (η_p_^2^) as the effect size for the ANOVA effects. Pairwise effect size (ES) is also reported where appropriate and interpreted according to Cohen (1988) as trivial (<0.20), small (0.20–0.49), medium (0.50–0.79) and large (≥0.80). This effect size approach was also used for the exploratory sub‐group analysis. The use of ANOVA was avoided due to the small sample size of the sub‐groups.

For cardiopulmonary variables that featured iso‐time peak, day 1 and day 2 values were compared with participants at each corresponding measurement point, using a 2 (LC vs. CON) x 2 (day 1 vs. day 2) x 3 (pre vs. iso‐time vs. post) mixed ANOVA treatment groups (LC and CON) across repeated exercise testing. Treatment group was the between‐participant factor, while day (day 1 and day 2) and measurement time (rest, VT1 and iso‐time peak) were within‐participant factors. For VT1, day 1 and day 2 values were only compared among participants where VT1 was identifiable on both CPET days. No inferential comparison between rest and iso‐time peak was performed where VT1 could not be identified with sufficient confidence.

Missing data (<1% of observation), were addressed using multiple imputation on eight data points in a statistical software package (SPSS Statistics, IBM Corp., Armonk, NY USA). These were three BP measurements for three separate participants (systolic and diastolic) and two BF data point for two separate participants. The main findings were unchanged in observed‐case analyses. Statistical significance was accepted as *P *< 0.05. Data are presented as means ± standard deviation unless otherwise stated.

### 2.7 Subgroup analysis

Metabolic acidosis was defined as a pH of <7.35 au and a HCO_3_^−^ value <22 mmol L^−1^. Descriptive statistics are reported (i.e., mean ± standard deviation) alongside pairwise effect sizes. The Heges *g* was selected to account for the small sample bias and interpreted as per Cohen thresholds (Cohen, 1988). Inferential statistics were not conducted due to the small sample size, a lack of statistical power, and considering it was an explanatory hypothesis.

## 3 RESULTS

Pre‐COVID‐19 health status was similar between the long COVID and control cohorts; however, health status worsened for the long COVID cohort following infection(s) with SARS‐CoV‐2. The number of confirmed SARS‐CoV‐2 infections (mean ± SD) recorded was 2 ± 1 for both the long COVID and control cohorts.

### 3.1 Safety and tolerability

No severe adverse or adverse events were reported following undertaking the CPET on either protocol day. Three long COVID participants (10%) were unable to complete the full 12‐min protocol on both days due to fatigue experienced during the protocol. All control participants completed the full 12‐min protocol on both days.

### 3.2 Cardiopulmonary results

Cardiopulmonary measures at rest and iso‐time peak for CPET day 1 and day 2 for controls are presented in Table 2. Only one control participant had an identifiable VT1 across both days and as such no data for this threshold is presented in Table 2. No between‐day changes were observed at rest or iso‐time peak for controls.

**TABLE 2:** Cardiopulmonary measures at rest and iso‐time peak for CPET day 1 and 2 for control participants.

	Rest				Iso‐time peak			
Variable	Day 1	Day 2	*P*	Effect size	Day 1	Day 2	*P*	Effect size
${\dot{V}}_{O_{2}}$ (L min^−1^)	0.35 ± 0.09	0.33 ± 0.08	0.912	0.18	1.35 ± 0.16	1.31 ± 0.15	0.994	0.20
${\dot{V}}_{O_{2}}$ (mL kg^−1^ min^−1^)	5 ± 1	4 ± 1	1.000	0.14	18 ± 3	17 ± 3	1.000	0.25
${\dot{V}}_{E}/{\dot{V}}_{O_{2}}$ (AU)	23.1 ± 3.1	24.1 ± 4.2	1.000	0.19	26.1 ± 5.0	27.0 ± 4.6	1.000	0.17
${\dot{V}}_{E}/{\dot{V}}_{CO_{2}}$ (AU)	27.1 ± 3.1	28.0 ± 3.8	0.845	0.18	26.7 ± 3.0	27.7 ± 3.3	0.876	0.17
RER	0.84 ± 0.05	0.86 ± 0.07	0.822	0.21	0.96 ± 0.08	0.97 ± 0.07	0.985	0.13
${\dot{V}}_{E}$ (L min^−1^)	10.5 ± 2.4	10.6 ± 2.5	1.000	0.01	39.4 ± 8.3	39.5 ± 8.0	0.973	0.02
BF (breaths min^−1^)	15 ± 3	17 ± 4	0.697	0.30	27 ± 7	27 ± 8	1.000	0.01
${\dot{V}}_{CO_{2}}$ (L min^−1^)	0.29 ± 0.07	0.28 ± 0.07	1.000	0.11	1.30 ± 0.19	1.27 ± 0.17	1.000	0.26
$P_{\mathrm{ET}O_{2}}$ (mmHg)	109 ± 5	112 ± 4	1.000	0.37	108 ± 6	109 ± 6	1.000	0.14
$P_{\mathrm{ETC}O_{2}}$ (mmHg)	33 ± 3	32 ± 3	0.279	0.27	38 ± 4	38 ± 4	1.000	0.02

*Note*: *n* = 18 controls (mean ± standard Deviation).

Abbreviations: BF, breathing frequency; $P_{\mathrm{ET}O_{2}}$ and $P_{\mathrm{ETC}O_{2}}$ denote end‐tidal oxygen and carbon dioxide, respectively; RER, respiratory exchange ratio; ${\dot{V}}_{CO_{2}}$, carbon dioxide production; ${\dot{V}}_{E}$, minute ventilation; ${\dot{V}}_{E}/{\dot{V}}_{CO_{2}}$, the ventilatory equivalent for carbon dioxide; ${\dot{V}}_{E}/{\dot{V}}_{O_{2}}$, the ventilatory equivalent for oxygen; ${\dot{V}}_{O_{2}}$, oxygen uptake.

Cardiopulmonary measures at rest, VT1 and iso‐time peak on CPET day 1 and day 2 are presented in Table 3. A 3% decrease in ${\dot{V}}_{E}/{\dot{V}}_{O_{2}}$ was observed for long COVID at rest on day 2 compared with day 1 (*P* = 0.033). VT1 was identifiable for 19 (63%) and 16 participants (53%) on CPET day 1 and day 2, respectively. Here, VT1 was identifiable for 13 participants (43%) across both days. No between‐day changes were observed at VT1 or iso‐time peak for the long COVID group.

**TABLE 3:** Cardiopulmonary measures at rest, VT1, and iso‐time peak for CPET day 1 and 2 for long COVID participants.

	Rest				VT1 (*n* = 13)		Iso‐time peak			
Variable	Day 1	Day 2	*P* value	Effect size	Day 1	Day 2	Day 1	Day 2	*P* value	Effect size
${\dot{V}}_{O_{2}}$ (L min^−1^)	0.32 ± 0.08	0.33 ± 0.07	0.159	0.24	0.67 ± 0.17	0.63 ± 0.10	1.31 ± 0.16	1.31 ± 0.18	1.000	0.29
${\dot{V}}_{O_{2}}$ (mL kg^−1^ min^−1^)	4 ± 1	4 ± 1	0.444	0.17	9 ± 2	8 ± 1	16 ± 2	16 ± 3	1.000	0.20
${\dot{V}}_{E}/{\dot{V}}_{O_{2}}$ (AU)	25.4 ± 3.9	24.7 ± 3.8	**0.033** ^*^	0.54	22.1 ± 1.9	22.0 ± 2.0	29.4 ± 7.4	29.7 ± 7.2	0.911	0.13
${\dot{V}}_{E}/{\dot{V}}_{CO_{2}}$ (AU)	28.4 ± 3.4	27.9 ± 3.2	1.000	0.13	27.5 ± 2.2	27.7 ± 2.7	30.0 ± 5.6	30.3 ± 5.4	1.000	0.05
RER	0.89 ± 0.08	0.89 ± 0.07	1.000	0.05	0.80 ± 0.05	0.80 ± 0.05	0.97 ± 0.08	0.97 ± 0.08	1.000	0.01
${\dot{V}}_{E}$ (L min^−1^)	10.2 ± 2.6	10.6 ± 2.3	0.530	0.08	17.7 ± 4.6	17.0 ± 3.2	42.9 ± 12.5	43.0 ± 11.3	1.000	0.18
BF (breaths min^−1^)	14 ± 5	15 ± 4	1.000	0.08	19 ± 5	19 ± 5	28 ± 9	28 ± 9	1.000	0.05
${\dot{V}}_{CO_{2}}$ (L min^−1^)	0.28 ± 0.07	0.30 ± 0.07	0.141	0.22	0.54 ± 0.16	0.50 ± 0.09	1.27 ± 0.18	1.27 ± 0.18	0.832	0.22
$P_{\mathrm{ET}O_{2}}$ (mmHg)	111 ± 5	111 ± 3	1.000	0.18	104 ± 4	103 ± 4	110 ± 6	110 ± 6	1.000	0.18
$P_{\mathrm{ETC}O_{2}}$ (mmHg)	33 ± 3	33 ± 2	1.000	0.15	37 ± 3	37 ± 3	37 ± 5	37 ± 5	0.901	0.15

*Note*: *n* = 30 people living with long COVID (mean ± standard deviation). ^*^Significant difference (*P *< 0.05).

Abbreviations: BF, breathing frequency; $P_{\mathrm{ET}O_{2}}$ and $P_{\mathrm{ETC}O_{2}}$ denote end‐tidal oxygen and carbon dioxide, respectively; RER, respiratory exchange ratio; ${\dot{V}}_{CO_{2}}$, carbon dioxide production; ${\dot{V}}_{E}$, minute ventilation; ${\dot{V}}_{E}/{\dot{V}}_{CO_{2}}$, the ventilatory equivalent for carbon dioxide; ${\dot{V}}_{E}/{\dot{V}}_{O_{2}}$, the ventilatory equivalent for oxygen; ${\dot{V}}_{O_{2}}$, oxygen uptake; VT1, ventilatory threshold 1.

### 3.3 Acid–base balance

No significant time × treatment interaction was observed for pH (*P* = 0.582, η_p_^2^ = 0.033). There was a main effect for time (<0.001, η_p_^2^ = 0.356) whereby pH was higher at rest versus post‐exercise for CPET day 1 in the LC group only (*P* = 0.014, mean difference (MD) = 0.04 au, ES = 1.25; Table 4). Similarly, no significant time × treatment interaction was observed for HCO_3_^−^ (*P* = 0.854, η_p_^2^ = 0.007), although a main effect for time was observed (*P *< 0.001, η_p_^2^ = 0.523) whereby HCO_3_^−^ was higher at rest versus post‐exercise for CPET day 1 in the LC (MD = 1.9 mmol L^−1^, *P* = 0.006, ES = 0.72) and CON groups (MD = 2.3 mmol L^−1^, *P* = 0.014, ES = 0.94). HCO_3_^−^ was lower post‐exercise compared to rest for CPET day 2 in the CON group (MD = 2.5 mmol L^−1^, *P* = 0.005, ES = 0.95). There was no time × treatment interaction for anion gap (*P* = 0.498, η_p_^2^ = 0.036); however, there was a main effect for time (*P *< 0.001, η_p_^2^ = 0.595) where the anion gap was greater between rest and post‐exercise for CPET day 1 for both LC (MD = 3 au, *P* = 0.004, ES = 1.16) and CON (MD = 3 au, *P* = 0.042, ES = 1.15). The anion gap was also greater from rest to post‐exercise for CPET day 2 for LC (MD = 3 au, *P* = 0.042, ES = 1.16). Similarly, K^+^ showed no time × treatment interaction (*P* = 0.159, η_p_^2^ = 0.091) but a main effect for time (*P *< 0.001, η_p_^2^ = 0.448). K^+^ was higher at post‐exercise on CPET day 1 compared to rest for LC (MD = 0.2 mmol L^−1^, *P* = 0.020, ES = 0.30), and CON (MD = 0.4 mmol L^−1^, *P* = 0.003, ES = 1.31). On CPET day 2 for the CON group, post‐exercise K^+^ was also greater compared to rest (MD = 0.2 mmol L^−1^, *P* = 0.029, ES = 0.77). There were no time × treatment interactions or main effects for Na^+^, iCa^2+^, Cl^−^, $P_{aO_{2}}$ or $P_{\mathrm{aC}O_{2}}$ (all *P *> 0.05; Table 4). Finally, there was no time × treatment interaction for lactate (*P* = 0.950, η_p_^2^ = 0.136); there was a main effect for time (*P *< 0.001, η_p_^2^ = 0.515). On day 1, CPET, lactate was greater at post‐exercise for the LC group compared to pre‐exercise (MD = 0.7 mmol L^−1^, *P* = 0.011, ES = 0.94).

**TABLE 4:** Blood acid–base balance variables from CPET1 and CPET2 for the long COVID (LC) and control (CON) groups.

Time point	Group	pH (au)	Bicarbonate (mmol L^−1^)	Lactate (mmol L^−1^)	Sodium (mmol L^−1^)	Calcium (mmol L^−1^)	Potassium (mmol L^−1^)	Chloride (mmol L^−1^)	Anion gap (au)	$P_{aO_{2}}$ (kPa)	$P_{\mathrm{aC}O_{2}}$ (kPa)
Rest day 1	LC CPET1 CON CPET1	7.41 ± 0.02 7.40 ± 0.02	24.6 ± 2.0 25.4 ± 1.9	1.3 ± 0.5^*^ 1.3 ± 0.5	140 ± 2 139 ± 2	1.23 ± 0.04 1.20 ± 0.03	4.6 ± 0.7 4.0 ± 0.3	109 ± 2 109 ± 2	5 ± 2 4 ± 2	9.5 ± 1.4 8.7 ± 1.3	5.2 ± 0.5 5.5 ± 0.6
Post‐exercise day 1	LC CPET1 CON CPET1	7.37 ± 0.04 7.37 ± 0.04	22.7 ± 3.1 23.1 ± 2.8	3.1 ± 1.9 2.0 ± 0.9	140 ± 4 139 ± 3	1.22 ± 0.07 1.21 ± 0.03	4.8 ± 0.6 4.4 ± 0.3	109 ± 2 109 ± 2	8 ± 3 7 ± 3	10.3 ± 1.3 11 ± 0.9	5.2 ± 0.8 5.3 ± 0.5
Rest day 2	LC CPET2 CON CPET2	7.39 ± 0.03 7.40 ± 0.02	24.5 ± 2.7 25.3 ± 2.1	1.4 ± 0.6 1.5 ± 0.5	139 ± 2 138 ± 2	1.21 ± 0.10 1.20 ± 0.02	4.4 ± 0.5 4.2 ± 0.3	109 ± 2 109 ± 2	5 ± 2 4 ± 2	9.3 ± 1.8 9.1 ± 1.5	5.4 ± 0.8 5.4 ± 0.5
Post‐exercise 2	LC CPET2 CON CPET2	7.38 ± 0.04 7.38 ± 0.04	22.6 ± 2.6 23.0 ± 2.6	3.3 ± 2.3 1.9 ± 0.9	140 ± 2 138 ± 3	1.23 ± 0.02 1.20 ± 0.03	4.8 ± 0.6 4.4 ± 0.2	110 ± 3 109 ± 2	8 ± 3 6 ± 3	10.8 ± 2 10.7 ± 1.4	5.1 ± 0.6 5.1 ± 0.5
ANOVA analysis	Time	*P *< 0.001, η_p_^2^ = 0.356	*P *< 0.001, η_p_^2^ = 0.523	*P *< 0.001, η_p_^2^ = 0.515	*P* = 0.831, η_p_^2^ = 0.011	*P* = 0.511, η_p_^2^ = 0.036	*P *< 0.001, η_p_^2^ = 0.448	*P* = 0.960, η_p_^2^ = 0.003	*P *< 0.001, η_p_^2^ = 0.595	*P* = 0.056; η_p_^2^ = 0.470	*P* = 0.383; η_p_^2^ = 0.217
	Treatment × time	*P* = 0.582, η_p_^2^ = 0.033	*P* = 0.854, η_p_^2^ = 0.007	*P* = 0.095, η_p_^2^ = 0.136	*P* = 0.374, η_p_^2^ = 0.056	*P* = 0.448, η_p_^2^ = 0.042	*P* = 0.159, η_p_^2^ = 0.091	*P* = 0.992, η_p_^2^ = 0.001	*P* = 0.498, η_p_^2^ = 0.036	*P* = 0.670; η_p_^2^ = 0.112	*P* = 0.597; η_p_^2^ = 0.140

All data presented as means ± standard deviation. ^*^Significant difference (*P *< 0.05). Abbreviations: au = arbitrary units; η_p_^2^, partial eta squared; $P_{\mathrm{aC}O_{2}}$, arterial partial pressure of carbon dioxide; $P_{aO_{2}}$, arterial partial pressure or oxygen.

### 3.4 Subgroup analysis

The subgroup analysis is presented based on subgroup‐defining characteristics rather than as independent outcomes, and a between‐group difference is not interpreted as evidence of an independent effect of LC and is rather an exploratory hypothesis.

In total, reported metabolic acidosis in the LC subgroup (LC‐S) was observed by *n *= 5/30 (17%) on CPET day 1 and *n *= 10/30 (33%) on CPET day 2 (Table 5). Three out of the five participants that presented metabolic acidosis on CPET day 1 presented this again on CPET day 2. In the CON group, metabolic acidosis was identified for *n *= 1/15 (7%) on CPET day 1 and *n *= 2/15 (13%) on CPET day 2.

**TABLE 5:** Subgroup analysis of blood acid–base balance variables from CPET1 and CPET2 for the long COVID subgroup (LC‐S) and control group (CON).

Time point	Group	pH (au)	Bicarbonate (mmol L^−1^)	Lactate (mmol L^−1^)	Sodium (mmol L^−1^)	Calcium (mmol L^−1^)	Potassium (mmol L^−1^)	Chloride (mmol L^−1^)	Anion gap (au)	$P_{aO_{2}}$ (kPa)	$P_{\mathrm{aC}O_{2}}$ (kPa)
Rest day 1	LC CPET1 (*n* = 5) CON CPET1	7.39 ± 0.01^a^ 7.40 ± 0.02	22.5 ± 1.9^c^ 25.4 ± 1.8	1.5 ± 0.4^b^ 1.3 ± 0.5	142 ± 4^c^ 139 ± 2	1.24 ± 0.02^c^ 1.20 ± 0.02	4.3 ± 0.4^c^ 4.0 ± 0.3	111 ± 2^c^ 109 ± 2	6 ± 2^a^ 4 ± 3	9.4 ± 1.7^b^ 8.7 ± 1.3	5.0 ± 0.5^c^ 5.5 ± 0.6
Post‐exercise day 1	LC CPET1 (*n* = 5) CON CPET1	7.34 ± 0.03^a^ 7.37 ± 0.04	19.1 ± 2.7^c^ 23.1 ± 2.7	4.8 ± 1.2^c^ 2.0 ± 0.9	140 ± 1^b^ 139 ± 3	1.19 ± 0.16^b^ 1.21 ± 0.03	4.5 ± 0.1^c^ 4.2 ± 0.3	111 ± 1^c^ 109 ± 2	9 ± 3^a^ 7 ± 3	10.3 ± 1.3^a^ 11 ± 0.9	4.7 ± 0.4^c^ 5.3 ± 0.5
Rest day 2	LC CPET2 (*n* = 10) CON CPET2	7.39 ± 0.02^b^ 7.40 ± 0.02	23.2 ± 1.7^c^ 25.4 ± 1.8	1.3 ± 0.4^b^ 1.5 ± 0.5	140 ± 3^c^ 138 ± 2	1.18 ± 0.17^b^ 1.20 ± 0.02	4.4 ± 0.4^a^ 4.2 ± 0.3	110 ± 2^a^ 109 ± 2	5 ± 1^b^ 4 ± 3	9.0 ± 1.1 9.1 ± 1.5	5.2 ± 0.5^b^ 5.4 ± 0.5
Post‐exercise day 2	LC CPET2 (*n* = 10) CON CPET2	7.34 ± 0.04^c^ 7.38 ± 0.04	19.8 ± 1.9^c^ 23.0 ± 2.5	5.9 ± 2.2^c^ 1.9 ± 0.9	140 ± 2^c^ 138 ± 2	1.24 ± 0.03^c^ 1.20 ± 0.02	4.8 ± 0.4^c^ 4.4 ± 0.2	110 ± 2^a^ 109 ± 2	10 ± 3^c^ 6 ± 3	10.3 ± 1.3^b^ 10.7 ± 1.4	5.0 ± 0.7 5.1 ± 0.5

All data presented as means ± standard deviation.

^a^ Medium effect size.

^b^ small effect size.

^c^ large effect size: versus control at the same corresponding time point. Abbreviations: au, arbitrary units; $P_{\mathrm{aC}O_{2}}$, arterial partial pressure of carbon dioxide; $P_{aO_{2}}$, arterial partial pressure or oxygen.

As expected, given the subgroup definition on CPET day 1 between the LC‐S subgroup and CON group, pH was lower in the LC‐S subgroup post‐exercise (MD = 0.03 au; *g* = 0.75; Figure 1). For CPET day 2 post‐exercise, pH was lower in the LC‐S subgroup than in the CON group (MD = 0.04 au; *g* = 0.97). HCO_3_^−^ was lower at pre‐exercise for LC‐S on CPET day 1 (MD = 2.9 mmol L^−1^; *g* = 1.54) and post‐exercise compared to CON (MD = 4.0 mmol L^−1^; *g* = 1.43; Figure 2). This was similar to CPET day 2, where HCO_3_^−^ was also lower at pre‐exercise (MD = 2.2 mmol L^−1^; *g* = 1.21) and post‐exercise (MD = 3.2 mmol L^−1^; *g* = 1.34) for the LC‐S subgroup compared to the CON group. For the additional metabolic variables examined on both CPET days 1 and 2, post‐exercise K^+^ was greater for LC‐S compared to CON (day 1, MD = 0.3 mmol L^−1^, *g* = 1.05; day 2, MD = 0.4 mmol L^−1^, *g* = 1.37). Correspondingly, the post‐exercise anion gap was greater for LC‐S compared to CON at both CPET day 1 and 2 (day 1, MD = 2 au, *g* = 0.64; day 2, MD = 4 au, *g* = 1.3; Figure 3). Finally, post‐exercise lactate for both CPET day 1 (MD = 2.8 mmol L^−1^; *g* = 2.81) and 2 (MD = 4.0 mmol L^−1^; *g* = 2.65) was greater for LC‐S compared to CON (Figure 4).

**FIGURE 1:**
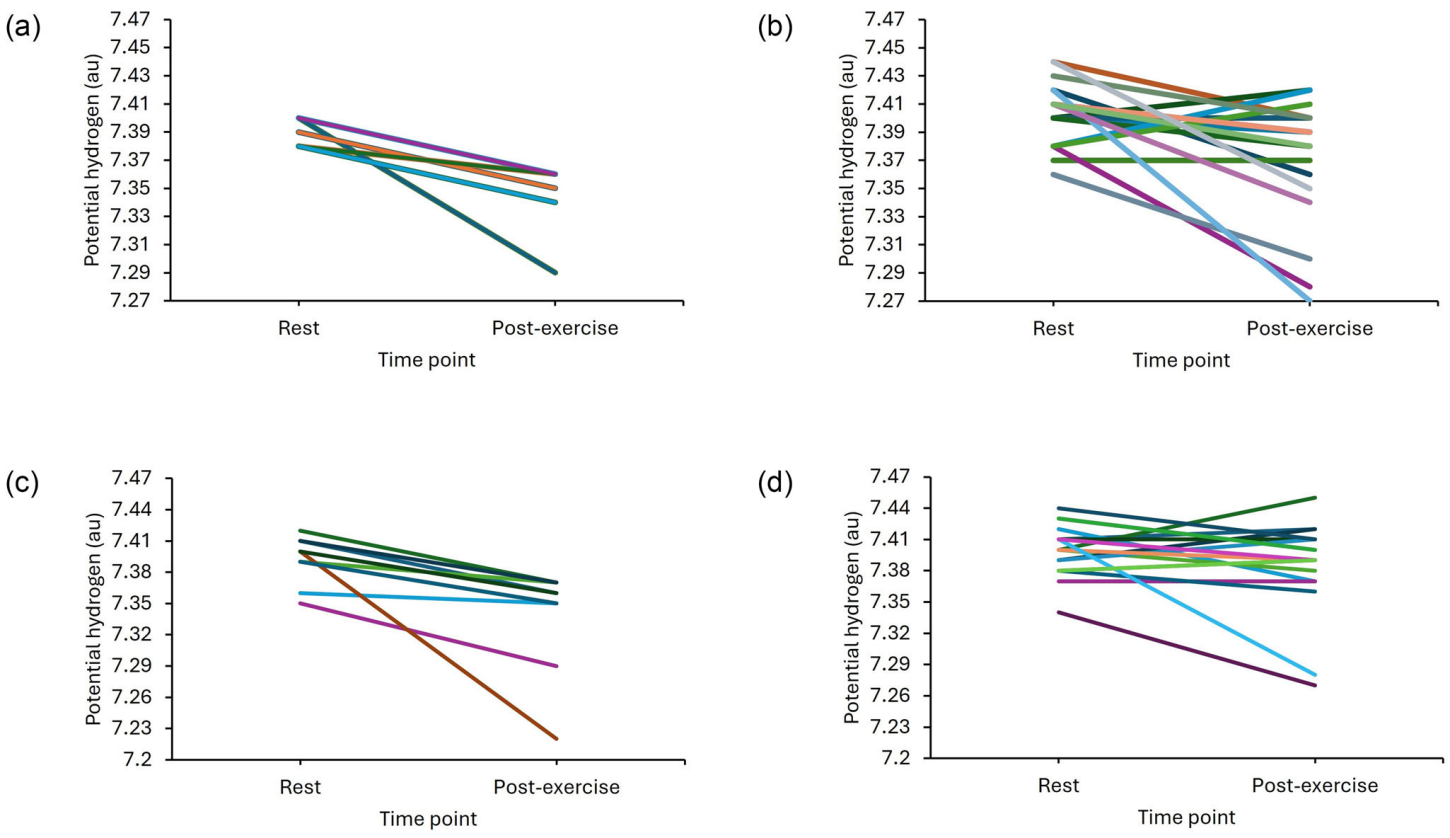
pH subgroup analysis. Individual lines represent individual data. (a) CPET1 LC; (b) CPET1 CON; (c) CPET2 LC; (d) CPET2 CON; CON, control; LC, long covid.

**FIGURE 2:**
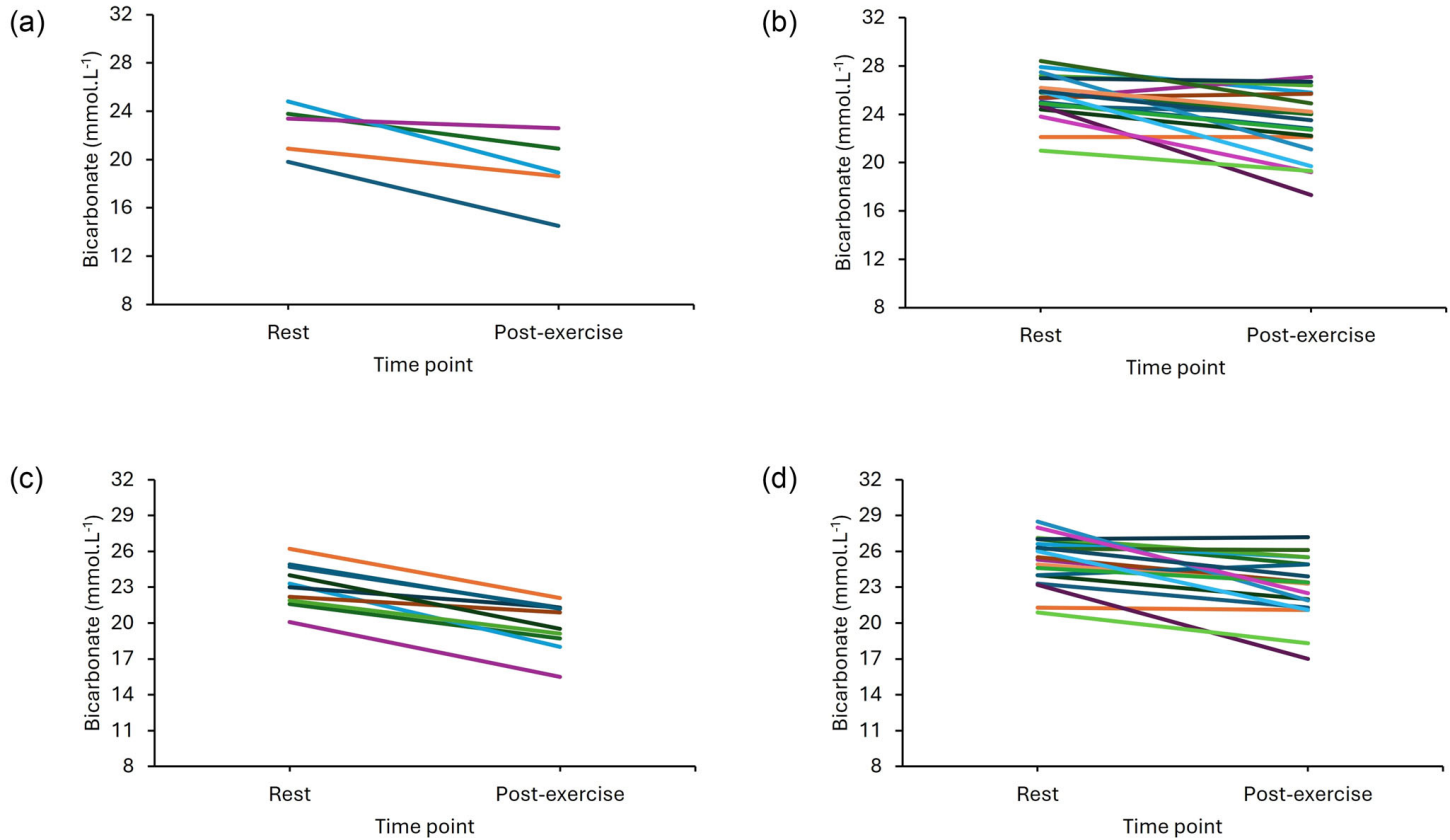
Bicarbonate (HCO_3_^−^) subgroup analysis. Individual lines represent individual data. (a) CPET1 LC; (b) CPET1 CON; (c) CPET2 LC; (d) CPET2 CON; CON, control; LC, long covid.

**FIGURE 3:**
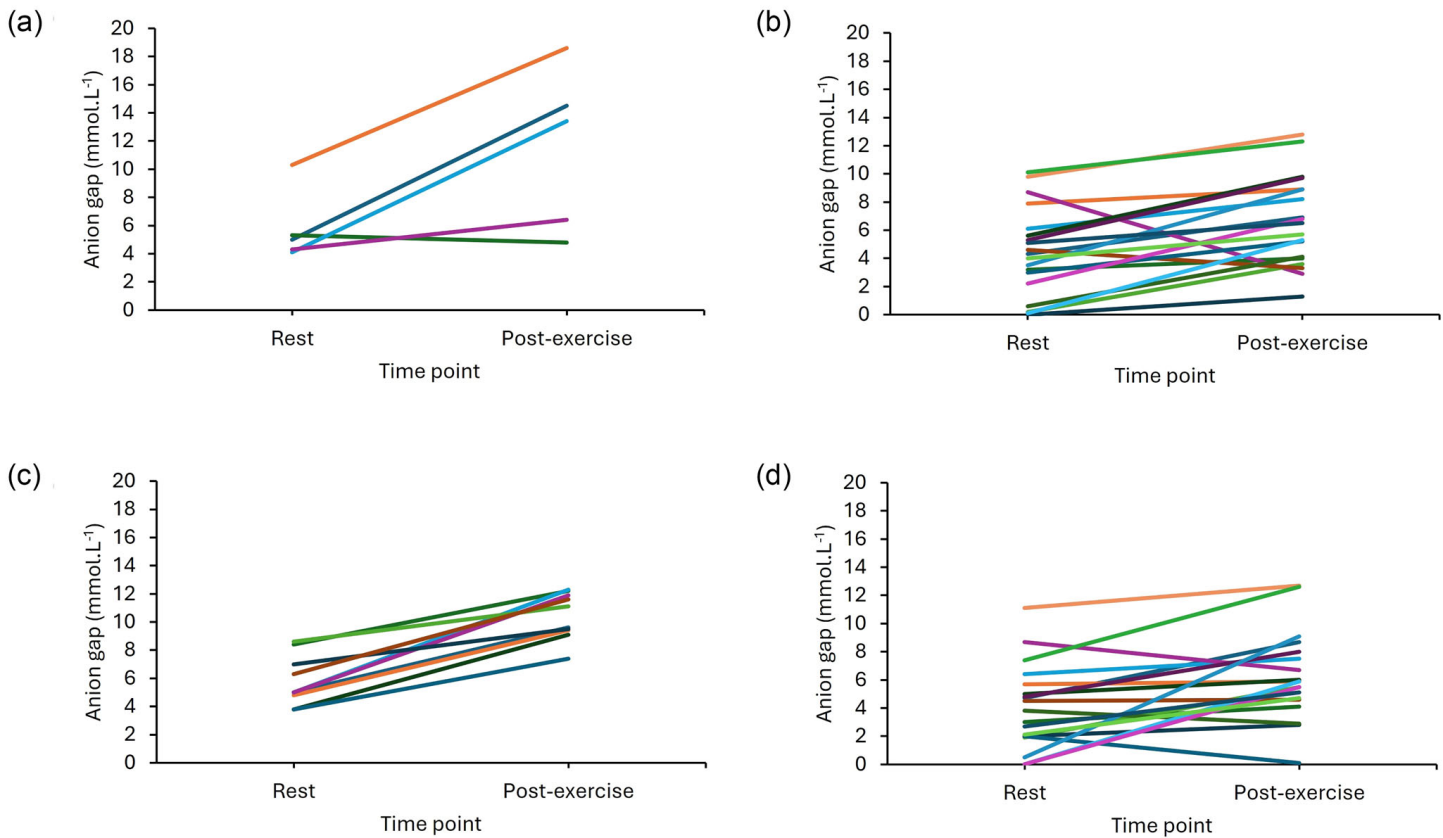
Anion gap subgroup analysis. Individual lines represent individual data. (a) CPET1 LC; (b) CPET1 CON; (c) CPET2 LC; (d) CPET2 CON; CON, control; LC, long covid.

**FIGURE 4:**
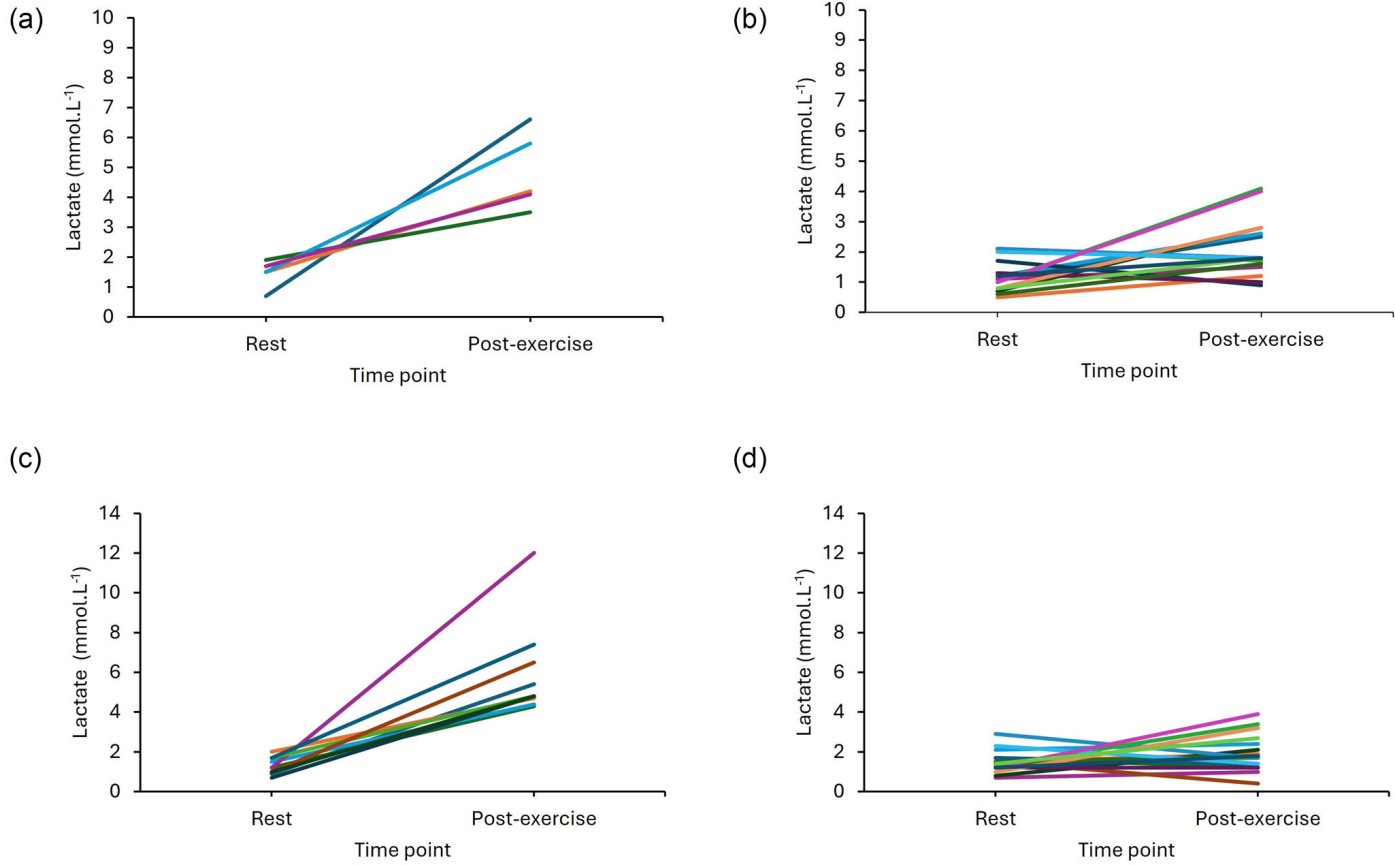
Lactate subgroup analysis. Individual lines represent individual data. (a) CPET1 LC; (b) CPET1 CON; (c) CPET2 LC; (d) CPET2 CON; CON, control; LC, long covid.

## 4 DISCUSSION

This study aimed to characterise acid–base balance responses to submaximal exercise in people living with LC. Across the LC group as a whole, there were no significant treatment differences in cardiorespiratory and acid–base balance across the two days of sub‐maximal exercise testing. This suggests that there are no limitations on these two areas of metabolism in people living with LC. The magnitude and pattern of this response did vary between individuals, however, such that the exploratory subgroup analysis showed some individuals with LC following submaximal exercise on day 1 and day 2 (17% and 33%, respectively) exhibited a more pronounced metabolic acidosis response compared with matched controls. Importantly, this occurred in the absence of cardiorespiratory responses that would typically be associated with significant disturbances in acid–base homeostasis, indicating an atypical physiological response to exertion for a meaningful proportion of those in the LC group. Equally, this study excluded participants with moderate–severe risk of PEM, and therefore these phenomena may be even more prominent if those with more severe post‐exertional limitations were included. Such disproportionate metabolic stress at relatively low exercise intensities may contribute to the reduced exercise tolerance commonly reported in some of this population, which requires further research.

There was no statistically significant effect on acid–base balance or lactate concentration across the whole group in the current study. However, with 17% and 33% of the LC cohort on CPET day 1 and day 2 demonstrating disturbance to acid–base balance, this suggests that changes beyond the whole group response should be considered. The current study findings are akin to Lien et al. (2019) who reported that lactate was abnormally higher within a group of myalgic encephalomyelitis/chronic fatigue syndrome (ME/CFS) females during repeated CPET testing compared to a control group. Given that exacerbated lactate also leads to concomitant increases in H^+^ (Gough et al., 2017), this may explain the observed metabolic acidosis in the subgroup analysis. This may subsequently explain, at least in part, some cases of PEM following exercise and/or repeated exercise. It is worth noting, however, that the response in ME/CFS patients seems to be more consistent across the whole group, which is likely explained by the greater variability in symptoms of LC compared to ME/CFS. This would be particularly important if one of the management/treatment strategies being considered by the clinician is exercise, which is common in current practice. Whilst Nantakool et al. (2024) reported that exercise rehabilitation improved LC cardiorespiratory fitness using a meta‐analysis approach, this likely fails to acknowledge the nuance of LC individuals’ pathology and those who present with acid–base balance disturbance. The severity of acid–base disturbance experienced by the LC subgroup in this study was substantial despite exercising only at submaximal intensities. Accordingly, these data suggest caution should be given towards involving people with LC in exercise and that maximal exercise testing may pose an exacerbated risk to those with acid–base disturbances.

Subgroup analysis highlighted some further concerns in respect of acid–base balance, where the subgroup mean HCO_3_^−^ of ∼19 mmol L^−1^ is reflective of exhaustive exercise in other maximal exercise studies (Lien et al., 2019). Considering the control group mean was ∼24 mmol L^−1^, this suggests a larger acid–base balance disturbance may be occurring in some people with LC and this warrants further investigation with larger cohorts. Whilst it could be argued that HCO_3_^−^ is a narrow view of acid–base balance, the anion gap, pH, and K^+^ fluxes also suggest a larger acid–base balance may be present in some people with LC in the present study. These changes occur despite the work rate of CPETs being similar between groups and may explain why reduced exercise performance elsewhere is commonly observed in LC individuals (Jamieson et al., 2024), as these changes in acid–base balance may exacerbate fatigue (Gough et al., 2017). This would be explained by the greater anaerobic energy contribution for the same given exercise in LC individuals compared to controls, which has a finite capacity compared to aerobic sources. This highlights an avenue for future exploratory work to confirm these preliminary subgroup findings. At this time, we suggest caution in the confirmation of the exploratory hypothesis until further data are available.

Importantly, the frequency of acid–base balance disturbances in the present study for LC increased following CPET2 compared to CPET1, which is similar to findings in ME/CFS participants (Lien et al., 2019). This may explain, at least in part, why PEM occurs in some LC individuals. These data would also provide a possible explanation for the common reports of difficulty sustaining exercise chronically (i.e., multiple exercise sessions per week) from LC individuals. For example, Wright et al. (2022) reported that adults with LC reported reduced physical activity compared with their pre‐COVID levels (27 vs. 360 min week^−1^). It may also explain the anecdotal reports of people with LC being unable to complete exercises they could do before the SARS‐CoV‐2 infection that initiated their LC status, as significant acid–base balance disturbance would lead to the earlier onset of fatigue. These observations suggest that the exercise intolerance observed in some LC individuals is not simply due to deconditioning but likely reflects underlying physiological disturbances, such as impaired acid–base balance and energy metabolism. Charlton et al. (2026) observed key differences between those with either LC or ME/CFS compared to age‐ and sex‐matched healthy controls who undertook strict 60‐day best rest. Bed rest induced muscle atrophy in healthy controls, something which was not observed in those with LC or ME/CFS. Instead, these two groups had fewer capillaries and more glycolytic fibres in skeletal muscle compared to healthy controls. This study indicated that whilst aerobic capacity may be similar between those who are deconditioned and LC, distinct skeletal muscle characteristics exist between the groups, indicating that physical inactivity alone does not cause the reduced exercise capacity in LC. These findings warrant further exploration to assess the potential link between acid–base balance disturbance from exercise, diminished exercise capacity, and/or exacerbated fatigue responses.

Across CPET day 1 and day 2, for both LC and CON, the group mean responses exhibited the expected transition from rest to iso‑time peak for ${\dot{V}}_{O_{2}}$ (absolute and relative), ${\dot{V}}_{CO_{2}}$, RER, ${\dot{V}}_{E}$ and breathing frequency, with no meaningful time × treatment interactions, indicating broadly preserved cardiopulmonary responses to submaximal exertion in LC at the whole‑group level. This pattern is consistent with prior syntheses showing that, while average peak ${\dot{V}}_{O_{2}}$ is often lower in LC than in non‑LC comparators, conventional ventilatory indices and gas‑exchange patterns are frequently within ‘normal’ limits or only modestly perturbed, implying that limitations may not be primarily ventilatory or haemodynamic in origin (Stringer et al., 2023; Durstenfeld et al., 2022). Moreover, day‑to‑day variability in core CPET metrics is generally small (<5%) under standardised conditions, supporting the reliability of the null interaction effects observed here. In contrast to the group‑mean picture, the LC subgroup with metabolic acidosis demonstrated clear abnormalities across both test days, despite comparable external work: lower iso‑time pH on day 1 and day 2, reduced HCO_3_^−^ at rest and iso‑time on both days and an increased anion gap. These changes occurred with markedly elevated iso‑time lactate on both days, indicating a greater reliance on anaerobic metabolism and reduced buffering capacity in a subset of LC participants. This interpretation aligns with independent data in post‑acute COVID showing impaired fatty‑acid oxidation and higher lactate accumulation during exercise (De Boer et al., 2022). There is evidence of a bioenergetic shift toward glycolysis even at modest workloads and reports that LC patients disproportionately utilise carbohydrate at rest and during exercise, particularly among those with pronounced fatigue/PEM. Interestingly, metabolic acidosis was more prevalent on day 2 (*n* = 10) than on day 1 (*n* = 5); however, only three participants on day 1 also experienced metabolic acidosis on day 2. This suggests that subgroup classification was not stable across days, but the previous day of exercise may have predisposed the additional day 2 participants to a greater risk of metabolic acidosis. This pattern converges with two‑day CPET work showing day 2 decrements in oxygen consumption and workload at the anaerobic threshold in both ME/CFS and LC, changes that occur despite largely normal ventilatory/haemodynamic indices, which supports the use of repeat testing to interrogate post‑exertional symptomatology (Davenport et al., 2026). While the clinical literature notes that CPET variables are generally reproducible across days, there is growing recognition that performance and metabolic indices can deteriorate on day 2 in susceptible patients, a phenomenon increasingly attributed to PEM (Davenport et al., 2026). Taken together, the present exploratory subgroup findings provide a hypothetical mechanistic link between submaximal exercise, acid–base disturbance and post‑exertional symptom worsening, which warrants further research

The dissociation between preserved conventional CPET markers at the group level and pronounced acid–base disturbance in a clinically relevant subgroup underscores the potential risk of misclassification when relying on a single‑day CPET and ventilatory metrics alone. Existing LC CPET series and syntheses suggest that abnormalities may be downstream at the level of peripheral oxygen utilisation and metabolism (Charlton et al., 2026; Appelman et al., 2024), rather than primary ventilatory dysfunction. This resonates with our subgroup data and with reports that targeted, CPET‑guided interventions can modify symptoms despite near‑normal gas exchange. Given the accumulating evidence that two‑day protocols can unmask PEM‑related deterioration, and that LC may involve defective substrate utilisation and lactate handling, metabolic profiling is therefore an important consideration before prescribing or recommending any form of exercise. This is particularly important as some individuals with LC may incur disproportionate metabolic stress at workloads that would ordinarily be considered submaximal, raising potential safety concerns if exercise prescriptions are escalated without identifying such phenotypes.

### 4.1 Limitations

Several methodological and interpretative considerations warrant emphasis in contextualising the present findings. Much of the existing two‐day CPET literature in LC remains preliminary, with effect sizes varying according to protocol design, recovery intervals and the criteria used to confirm maximal effort; thus, replication using standardised approaches is essential. Additionally, LC is a markedly heterogeneous condition, with wide variability in symptom profile, comorbidities and illness trajectory, meaning that group‐mean responses may obscure clinically meaningful subphenotypes and strengthen the case for stratification by metabolic or physiological presentation in both research and clinical pathways. Although altered substrate utilisation and impaired lactate handling offer a coherent explanatory basis for the acid–base disturbances observed in the present study, more definitive mechanistic insight, such as determining the contribution of mitochondrial dysfunction or microvascular dysregulation, will require integration of CPET data with targeted biomarkers and longer‐term follow‐up. It is also acknowledged that the acid–base balance disturbance was only found within a small subgroup, and we cannot confirm there are independent effects of LC on the exacerbated acid–base balance metabolism, and therefore further work is needed with larger cohorts. Given the bioenergetic difference between sexes, such recruitment would also enable the opportunity for appropriately powered sex‐related difference to be assessed which could not be performed in the present study. These broader limitations intersect with those specific to the present study: our sample was predominantly white British, limiting generalisability across ethnic groups; the two‐day CPET protocol, while valid for assessing acute responses, does not capture the chronic or fluctuating nature of LC symptoms, meaning that potential longer‐term variations in cardiopulmonary or metabolic function remain uncharacterised; and although our total sample size was comparable to previous CPET studies, the subgroup experiencing metabolic acidosis was small, underscoring the need for larger, stratified investigations. Collectively, these considerations highlight the importance of cautious interpretation and the need for further work to validate and extend these preliminary observations.

### 4.2 Conclusion

This study reports that across a LC group there was no significant acid–base balance disturbance. However, some LC individuals display significant acid–base balance dysfunction that increases in prevalence with repeated submaximal exercise. These findings suggest that acid–base balance could form part of screening procedures, but future work must first independently validate the study results in larger cohorts. If verified, mitigation strategies should be explored for those who display significant acid–base balance disturbance to help alleviate the severity of disturbances and improve exercise tolerance. Future research should also provide consideration to cardiopulmonary and acid–base balance responses over a chronic time frame (i.e., days and weeks) and align these responses with reporting of PESE and PEM.

## AUTHOR CONTRIBUTIONS

All authors contributed to the Conception or design of the work. Data acquisition was conducted by all authors except Lewis A. Gough. Analysis, interpretation and drafting was conducted by Mark A Faghy, Callum Thomas, and Lewis A. Gough. All authors approved the final manuscript and agree to be accountable for all aspects of the work in ensuring that questions related to the accuracy or integrity of any part of the work are appropriately investigated and resolved; and all persons designated as authors qualify for authorship, and all those who qualify for authorship are listed.

## CONFLICT OF INTEREST

The authors declare no conflicts of interest.

## GENERATIVE AI STATEMENT

No AI was used for this study.

## Data Availability

The data that support the findings of this study are available on request from the corresponding author. The data are not publicly available due to privacy or ethical restrictions.

## References

[eph70539-bib-0001] Agarwala, P. , & Salzman, S. H. (2020). Six‐minute walk test: Clinical role, technique, coding, and reimbursement. Chest, 157(3), 603–611. 10.1016/j.chest.2019.10.014 PMC7609960 31689414

[eph70539-bib-0002] Alfano, G. , Fontana, F. , Mori, G. , Giaroni, F. , Ferrari, A. , Giovanella, S. , Ligabue, G. , Ascione, E. , Cazzato, S. , Ballestri, M. , Di Gaetano, M. , Meschiari, M. , Menozzi, M. , Milic, J. , Andrea, B. , Franceschini, E. , Cuomo, G. , Magistroni, R. , Mussini, C. , … Pinti, M. (2022). Acid base disorders in patients with COVID‐19. International Urology and Nephrology, 54(2), 405–410. 10.1007/s11255-021-02855-1 PMC8193956 34115260

[eph70539-bib-0003] Al‐Aly, Z. , Davis, H. , Mccorkell, L. , Soares, L. , Wulf‐Hanson, S. , Iwasaki, A. , & Topol, E. J. (2024). Long COVID science, research and policy. Nature Medicine, 30(8), 2148–2164. 10.1038/s41591-024-03173-6 39122965

[eph70539-bib-0004] Appelman, B. , Charlton, B. T. , Goulding, R. P. , Kerkhoff, T. J. , Breedveld, E. A. , Noort, W. , Offringa, C. , Bloemers, F. W. , Van Weeghel, M. , Schomakers, B. V. , Coelho, P. , Posthuma, J. J. , Aronica, E. , Joost Wiersinga, W. , Van Vugt, M. , & Wüst, R. C. I. (2024). Muscle abnormalities worsen after post‐exertional malaise in long COVID. Nature Communications, 15(1), 17. 10.1038/s41467-023-44432-3 PMC10766651 38177128

[eph70539-bib-0005] De Boer, E. , Petrache, I. , Goldstein, N. M. , Olin, J. T. , Keith, R. C. , Modena, B. , Mohning, M. P. , Yunt, Z. X. , San‐Millán, I. , & Swigris, J. J. (2022). Decreased fatty acid oxidation and altered lactate production during exercise in patients with post‐acute COVID‐19 syndrome. American Journal of Respiratory and Critical Care Medicine, 205(1), 126–129. 10.1164/rccm.202108-1903LE PMC8865580 34665688

[eph70539-bib-0006] Charlton, B. T. , Slaghekke, A. , Appelman, B. , Eggelbusch, M. , Huijts, J. Y. , Noort, W. , Hendrickse, P. W. , Bloemers, F. W. , Posthuma, J. J. , Van Amstel, P. , Goulding, R. P. , Degens, H. , Jaspers, R. T. , Van Vugt, M. , & Wüst, R. C. I. (2026). Skeletal muscle properties in long COVID and ME/CFS differ from those induced by bed rest. Nature Communications, 17(1), 9125. 10.1038/s41467-026-75725-y PMC13518852 42649155

[eph70539-bib-0007] Chiumello, D. , Pozzi, T. , Fratti, I. , Modafferi, L. , Montante, M. , Papa, G. F. S. , & Coppola, S. (2022). Acid‐base disorders in COVID‐19 patients with acute respiratory distress syndrome. Journal of Clinical Medicine, 11(8), 2093. 10.3390/jcm11082093 PMC9024702 35456186

[eph70539-bib-0008] Cohen, J. (1988). Statistical power analysis for the behavioral sciences. Routledge.

[eph70539-bib-0009] Cotler, J. , Holtzman, C. , Dudun, C. , & Jason, L. A. (2018). A brief questionnaire to assess post‐exertional malaise. Diagnostics, 8(3), 66. 10.3390/diagnostics8030066 PMC6165517 30208578

[eph70539-bib-0010] Curtis, K. J. , O'brien, K. A. , Tanner, R. J. , Polkey, J. I. , Minnion, M. , Feelisch, M. , Polkey, M. I. , Edwards, L. M. , & Hopkinson, N. S. (2015). Acute dietary nitrate supplementation and exercise performance in COPD: A double‐blind, placebo‐controlled, randomised controlled pilot study. PLoS ONE, 10(12), e0144504. 10.1371/journal.pone.0144504 PMC4689520 26698120

[eph70539-bib-0011] Davenport, T. E. , Stevens, S. R. , Stevens, J. , & Van Ness, J. M. (2026). ME/CFS and long COVID demonstrate similar bioenergetic impairment and recovery failure on two‐day cardiopulmonary exercise testing. Clinical & Translational Metabolism, 24(1), 6.

[eph70539-bib-0012] De Vries, J. , Michielsen, H. , Van Heck, G. L. , & Drent, M. (2004). Measuring fatigue in sarcoidosis: The Fatigue Assessment Scale (FAS). British Journal of Health Psychology, 9(3), 279–291. 10.1348/1359107041557048 15296678

[eph70539-bib-0013] Durstenfeld, M. S. , Sun, K. , Tahir, P. , Peluso, M. J. , Deeks, S. G. , Aras, M. A. , Grandis, D. J. , Long, C. S. , Beatty, A. , & Hsue, P. Y. (2022). Use of cardiopulmonary exercise testing to evaluate long COVID‐19 symptoms in adults: A systematic review and meta‐analysis. JAMA Network Open, 5(10), e2236057. 10.1001/jamanetworkopen.2022.36057 PMC9557896 36223120

[eph70539-bib-0014] Faghy, M. A. , Sylvester, K. P. , Cooper, B. G. , & Hull, J. H. (2020). Cardiopulmonary exercise testing in the COVID‐19 endemic phase. BJA: British Journal of Anaesthesia, 125(4), 447–449. 10.1016/j.bja.2020.06.006 PMC7287473 32571569

[eph70539-bib-0015] Faghy, M. A. , Dalton, C. , Duncan, R. , Arena, R. , & Ashton, R. E. (2024). Using cardiorespiratory fitness assessment to identify pathophysiology in long COVID–Best practice approaches. Progress in Cardiovascular Diseases, 83, 55–61. 10.1016/j.pcad.2024.02.005 38417769

[eph70539-bib-0016] Gough, L. A. , Rimmer, S. , Osler, C. J. , & Higgins, M. F. (2017). Ingestion of sodium bicarbonate (NaHCO_3_) following a fatiguing bout of exercise accelerates postexercise acid‐base balance recovery and improves subsequent high‐intensity cycling time to exhaustion. International Journal of Sport Nutrition and Exercise Metabolism, 27(5), 429–438. 10.1123/ijsnem.2017-0065 28530505

[eph70539-bib-0017] Greenhalgh, T. , Sivan, M. , Perlowski, A. , & Nikolich, J. Ž. (2024). Long COVID: A clinical update. The Lancet, 404(10453), 707–724. 10.1016/S0140-6736(24)01136-X 39096925

[eph70539-bib-0018] Herdman, M. , Gudex, C. , Lloyd, A. , Janssen, M. F. , Kind, P. , Parkin, D. , Bonsel, G. , & Badia, X. (2011). Development and preliminary testing of the new five‐level version of EQ‐5D (EQ‐5D‐5L). Quality of Life Research, 20(10), 1727–1736. 10.1007/s11136-011-9903-x PMC3220807 21479777

[eph70539-bib-0019] Jamieson, A. , Al Saikhan, L. , Alghamdi, L. , Hamill Howes, L. , Purcell, H. , Hillman, T. , Heightman, M. , Treibel, T. , Orini, M. , Bell, R. , Scully, M. , Hamer, M. , Chaturvedi, N. , Montgomery, H. , Hughes, A. D. , Astin, R. , & Jones, S. (2024). Mechanisms underlying exercise intolerance in long COVID: An accumulation of multisystem dysfunction. Physiological Reports, 12(3), e15940. 10.14814/phy2.15940 PMC10861355 38346773

[eph70539-bib-0020] Larson, R. D. (2013). Psychometric properties of the modified fatigue impact scale. International Journal of MS Care, 15(1), 15–20. 10.7224/1537-2073.2012-019 PMC3883028 24453758

[eph70539-bib-0021] Lien, K. , Johansen, B. , Veierød, M. B. , Haslestad, A. S. , Bøhn, S. K. , Melsom, M. N. , Kardel, K. R. , & Iversen, P. O. (2019). Abnormal blood lactate accumulation during repeated exercise testing in myalgic encephalomyelitis/chronic fatigue syndrome. Physiological Reports, 7(11), e14138. 10.14814/phy2.14138 PMC6546966 31161646

[eph70539-bib-0022] Nantakool, S. , Sa‐Nguanmoo, P. , Konghakote, S. , & Chuatrakoon, B. (2024). Effects of exercise rehabilitation on cardiorespiratory fitness in long‐COVID‐19 survivors: A meta‐analysis. Journal of Clinical Medicine, 13(12), 3621. 10.3390/jcm13123621 PMC11204731 38930148

[eph70539-bib-0023] Nicolò, M. , Ferro Desideri, L. , Bassetti, M. , & Traverso, C. E. (2021). Hydroxychloroquine and chloroquine retinal safety concerns during COVID‐19 outbreak. International Ophthalmology, 41(2), 719–725. 10.1007/s10792-020-01593-0 PMC7499926 32946004

[eph70539-bib-0024] Owen, R. , Ashton, R. E. M. , Bewick, T. , Copeland, R. J. , Ferraro, F. V. , Kennerley, C. , Phillips, B. E. , Maden‐Wilkinson, T. , Parkington, T. , Skipper, L. , Thomas, C. , Arena, R. , Formenti, F. , Ozemek, C. , Veluswamy, S. K. , Gururaj, R. , & Faghy, M. A. (2025). Profiling the persistent and episodic nature of long COVID symptoms and the impact on quality of life and functional status: A cohort observation study. Journal of Global Health, 15, 04006. 10.7189/jogh.15.04006 PMC11801655 39913532

[eph70539-bib-0025] Pescatello, L. S. (Ed.). (2014). ACSM's guidelines for exercise testing and prescription. Lippincott Williams & Wilkins.

[eph70539-bib-0026] Sjöström, A. , Rysz, S. , Sjöström, H. , & Höybye, C. (2021). Electrolyte and acid‐base imbalance in severe COVID‐19. Endocrine Connections, 10(7), 805–814. 10.1530/EC-21-0265 PMC8346182 34156969

[eph70539-bib-0027] Stringer, W. W. , Heffernan, C. , Dineen, E. , Co Nguyen, L. , Gattoni, C. , Ferguson, C. , Lanks, C. W. , Abbasi, A. , & W Decato, T. (2023). Utility of 2‐day ardiopulmonary xercise testing protocol in long‐haul covid (LHC) patients: Preliminary data. Chest, 164(4), A5778–A5779.

[eph70539-bib-0028] Thaweethai, T. , Jolley, S. E. , Karlson, E. W. , Levitan, E. B. , Levy, B. , Mccomsey, G. A. , Mccorkell, L. , Nadkarni, G. N. , Parthasarathy, S. , Singh, U. , Walker, T. A. , Selvaggi, C. A. , Shinnick, D. J. , Schulte, C. C. M. , Atchley‐Challenner, R. , Horwitz, L. I. , Foulkes, A. S. , Aberg, J. A. , Adolphi, N. L. , … Zisis, S. (2023). Development of a definition of postacute sequelae of SARS‐CoV‐2 infection. Journal of the American Medical Association, 329(22), 1934. 10.1001/jama.2023.8823 PMC10214179 37278994

[eph70539-bib-0029] Thomas, C. , Kudiersky, N. , Ansdell, P. , Ashton, R. E. , Brown, C. , Bewick, T. , Carr, J. , Hume, E. , Spillane, P. , Pastorio, E. , Owen, R. , Maden‐Wilkinson, T. , Mcneil‐Angopa, E. , Parkington, T. , Arena, R. , Ozemek, C. , Formenti, F. , Veluswamy, S. K. , Gururaj, R. , & Faghy, M. A. (2026). Submaximal 2‐day cardiopulmonary exercise testing to assess exercise capacity and post‐exertional symptom exacerbation in people with long COVID. Experimental Physiology, 111(2), 435–448. 10.1113/EP092576 PMC12857497 40515424

[eph70539-bib-0030] Van Der Togt, V. , & Rossman, J. S. (2023). Hypothesis: Inflammatory acid‐base disruption underpins long covid. Frontiers in Immunology, 14, 1150105. 10.3389/fimmu.2023.1150105 PMC10140510 37122723

[eph70539-bib-0031] Wright, J. , Astill, S. L. , & Sivan, M. (2022). The relationship between physical activity and long COVID: A cross‐sectional study. International Journal of Environmental Research and Public Health, 19(9), 5093. 10.3390/ijerph19095093 PMC9105041 35564488

